# Correspondence—Original

**Published:** 1856-01

**Authors:** 


New York City, Jan. 1st, 1856.

Mr. Eeitor:—Sir, will you permit me through your Journal to make a few remarks on a new material which has been lately introduced as a base for artificial teeth, called *Slayton's Colored Gutta Percha.* Gutta percha has been used for this purpose for the last eight years in England and Germany, and for the last three years it has attracted some attention in this country, more however within the last six months than at any time previous.

Dr. Slayton of Madison, Indiana, commenced a course of experiments with this article, and succeeded in refining it, and giving it a permanent gum color. Gutta percha has been colored before, but the color was not durable. The article made by Dr. Slayton I have been using for the last three months, to my entire satisfaction. It is all that it is said to be. Dr. S. has offered it to the profession merely for *temporary work*, and advises all to try it for that purpose, before they recommend it for anything more durable. The claim is certainly a very *modest* and *honest* one. Any dentist who has become familiar with the working of this article, and can use it *properly*, will not dispense with it in his practice, if he uses it for nothing but temporary work. It is cheap and easy to work, and I have become so familiar with the working of it, that from six to eight hours is all the time it requires for me to finish a full set, and when it is finished, it looks as well, and fits better than any plate I can make. I know of a large number of dentists in this section of country that are using this material, and *nearly* all I believe are pleased with it, some cannot use it at all, while others are watching and waiting to see how others succeed, a little suspicious that there is a *mare's nest* somewhere. There is another class who condemn the whole thing as a *humbug*, without even examining into its merits, or testing it in any shape, but take it for granted, that anything which is new or differs in the least from the old way, must be a humbug.

Now, I suppose this is all right enough, they have a perfect *right* to do so, and they either mean much good to the profession, or they simply mean to attract attention to themselves, by showing what discerning minds they have in detecting a humbug at a glance, without seeing or testing its value. Ask them if they have seen and tested the improvement, and they quickly answer, "*No*, they have no occasion to use it," they do not stoop to such quackery, they are not in the habit of dealing in *humbugs*, and are surprised and mortified that men of good standing will be so *unprofessional* as to *test* or *even* countenance such *quackery*.

With all this devotion to the profession, I have *never* known any improvement or even an original idea to originate in their brain. They are the first to raise the warning voice, but always so *shape* their opposition, that if the thing should prove of real utility, and be received by the profession as an improvement, they can jump on and ride, and at the *same time* remind the originator how much he is indebted to them for his success. But on the other hand, if the thing should *fail* to be what is desired, they give the profession to understand they are *everlastingly* indebted to them for their timely warning, which has saved them from *disgrace*.

I have also noticed that this same class of dentists are always opposed to any improvement being *patented*. "It is unprofessional."

Ask such, if an author who has spent time, labor and money, is not justified in obtaining a copyright for his work, and they will tell you he is. Ask them if a mechanic who has spent years of hard toil, and impoverished himself and family, is not justified in obtaining a patent for his improvement, "Yes, this is all right too." Ask them again, if a dentist who has spent weeks, months, and even years, and at the same time expended hundreds of dollars in investigating a certain principle in dentistry, and at last been so fortunate as to make a *decided* improvement, if he ought not to take out a patent for his improvement, and thereby receive a compensation for his labors? What will be their answer? "By no means, It is unprofessional, it is quackery." He ought, they say, to throw it open to the whole profession, and if it is an improvement, let all be benefitted by it. I would ask here what makes the difference? And who has made this unprofessional? Is it those men who think that experiments are constantly elevating their profession, or is it they who have no ideas of their own, and wish to be benefitted by others' improvements without allowing any compensation for the same?

They tell you that the *honor* of the thing is sufficient compensation, but you will most generally find them the first to try to *rob* him of that honor, by saying it is nothing new, &c., that they have used it for years and have abandoned it. These persons generally do their work different from other dentists, "it is a way of their own," which no one but themselves know, and if you are willing to pay them a good price, they will instruct you. This I suppose is *professional.* Now, Mr. Editor, *what does* all this mean. Is this the proper way to elevate our profession? Carry the principle out, and where will it lead you? Show me the man that is willing to spend months, years, and hundreds of dollars to make an improvement, and then give it to the profession without any compensation, but merely for the honor, and I will bless the day that ushered that man into the world, and will contribute freely to raise a monument to his name.

Dentistry is yet in its infancy, notwithstanding the rapid strides it has made within the last few years. Every member of the profession feels that there ought to be many improvements made both in the operative and mechanical departments, before it will take the high position it is destined to do. These improvements will not happen by *chance*, but will be the result of much thought and hard *labor*, with the prospect of some little compensation for that *labor*. I am in favor of denouncing every thing that is proven to be a *humbug*, more especially where the *desire* is to *humbug*, but before we denounce a new thing let us test it well.

I also think that a dentist like other men should be rewarded for his labor, and I am willing to pay a reasonable price for any instruction from him, but at the *same time* to be held up before the world as dealing in quack nostrums, because I have seen fit to test an improvement condemned by others, is far from right. I have been led to make these few remarks by seeing a disposition to condemn a new thing before it has been tested. It is only by experimenting that we can ever expect to arrive at *perfection*. And he who tries even though he fail, merits the thanks of the whole profession, for a failure often leads to a still greater improvement. Gutta percha is, I feel satisfied, a great improvement, not that it is already perfected, but the starting point is made. Many will think and experiment, and in the end we shall have what we want. J. P. F.

[The foregoing article came to hand too late for insertion in its proper place with the original matter. We publish it however, as it expresses the sentiments of many in the profession. We do not believe that any known principle of science can be patented. It is only a *new mode* of applying such principles that can be patented.—Ed.]

